# Glucose-regulated protein 94 mediates progression and metastasis of esophageal squamous cell carcinoma via mitochondrial function and the NF-kB/COX-2/VEGF axis

**DOI:** 10.18632/oncotarget.24114

**Published:** 2018-01-10

**Authors:** Chien-Yu Huang, Chia-Hwa Lee, Chao-Chiang Tu, Chih-Hsiung Wu, Ming-Te Huang, Po-Li Wei, Yu-Jia Chang

**Affiliations:** ^1^ Department of Surgery, College of Medicine, Taipei Medical University, Taipei, Taiwan; ^2^ Division of General Surgery, Department of Surgery, Shuang Ho Hospital, Taipei Medical University, Taipei, Taiwan; ^3^ School of Medical Laboratory Science and Biotechnology, College of Medical Science and Technology, Taipei Medical University, Taipei, Taiwan; ^4^ Graduate Institute of Clinical Medicine, College of Medicine, Taipei Medical University, Taipei, Taiwan; ^5^ Division of General Surgery, Department of Surgery, Fu Jen Catholic University Hospital; School of Medicine, College of Medicine, Fu-Jen Catholic University, Taipei, Taiwan; ^6^ Division of Colorectal Surgery, Department of Surgery, Wan Fang Hospital, Taipei Medical University, Taipei, Taiwan; ^7^ Cancer Research Center and Translational Laboratory, Department of Medical Research, Taipei Medical University Hospital, Taipei Medical University, Taipei, Taiwan; ^8^ Division of Colorectal Surgery, Department of Surgery, Taipei Medical University Hospital, Taipei Medical University, Taipei, Taiwan; ^9^ Graduate Institute of Cancer Biology and Drug Discovery, Taipei Medical University, Taipei, Taiwan; ^10^ En Chu Kong Hospital, Taipei, Taiwan

**Keywords:** ESCC, GRP94, VEGF, COX-2

## Abstract

Esophageal cancer is a worldwide health problem with a very poor prognosis. Therefore, new diagnostic biomarkers or therapeutic strategies for identifying and managing esophageal squamous cell carcinoma (ESCC) are urgently needed. Glucose-regulated protein 94 (GRP94) is one of major endoplasmic reticulum-stress response proteins that plays a key role in cancer progression and therapeutic responses. However, the role of GRP94 in ESCC progression and metastasis remains unclear. The tissue array results indicated that higher GRP94 expression levels were associated with lower overall survival and higher lympho-node metastasis. Silencing GRP94 (GRP94-KD) reduced cell proliferation, migration and invasion in ESCC cells. In a xenotransplantation assay, silencing GRP94 reduced cell proliferation in the zebrafish embryo. Transmission electron microscopy revealed impaired mitochondria in GRP94-KD cells, which exhibited reduced basal respiration, spare respiratory capacity and ATP production and increased oxidative damage compared with scrambled control cells. Regarding the molecular mechanism underlying the effects of GRP94 knockdown, we found that silencing GRP94 may reduce the level of NF-kB, c-Jun, p38, IL-6, vascular endothelial growth factor (VEGF), and cyclooxygenase-2 (COX-2) as well as activation of AKT and ERK. In conclusion, our results indicate that silencing GRP94 in ESCC cells suppressed cancer growth and the metastatic potential via mitochondrial functions and NF-kB/COX-2/VEGF in ESCC cells.

## INTRODUCTION

Esophageal cancer is a very aggressive cancer comprising two main histological types, squamous cell carcinoma and adenocarcinoma. In certain countries with a high incidence of esophageal carcinoma such as China, up to 90% of all esophageal cancer cases are squamous cell carcinomas [1]. The risk factors for esophageal squamous cell carcinoma (ESCC) and esophageal adenocarcinoma are smoking, excessive alcohol intake, high-temperature drinks [2, 3], obesity, and chronic gastroesophageal reflux disease [4, 5]. The therapeutic outcomes of esophageal cancer are dismal, as most patients present with distant metastasis when first diagnosed. The 5-year survival rate ranges from 10%–20% and increases to 40% with surgical intervention [6–8]. Most patients with ESCC die within the first year after diagnosis, indicating the importance of early ESCC diagnosis. p53 gene mutations are frequently detected in esophageal cancer [9], and dysregulation of cell regulators, such as epidermal growth factor receptor [10, 11], HER-2/Neu [12], and vascular endothelial growth factor [13], has been investigated in ESCC. It is urgent to find an effective biomarker for improving prognostic and therapeutic efficacy in ESCC.

Glucose-related protein 94 (GRP94) is a member of the HSP90 family. GRP94 is an endoplasmic reticulum (ER) chaperone whose main function entails assisting with protein folding, assembly and secretion. GRP94 also helps cells to survive stresses, such as ischemia, starvation, radiation and chemotoxicity [14–17]. In malignant diseases, GRP94 is highly expressed in cancer tissues, and its expression correlates with the therapeutic response [16, 18]. Specifically, increased GRP94 protein expression is associated with poor tumor differentiation, invasiveness and metastasis, radioresistance and chemoresistance in many different cancers [15, 19, 20]. Silencing GRP94 expression promotes apoptosis with or without ER stress [21, 22], and blocking GRP94 function may suppress tumorigenesis and metastasis in liver cancer [23–25]. Immunohistochemical staining has demonstrated that there is a significant correlation between GRP94 expression and the progression of esophageal cancer [26–29]. However, the role and regulatory mechanism of GRP94 in ESCC progression is not fully understood.

Vascular endothelial growth factor (VEGF) is a pivotal factor in the complex process of angiogenesis. The VEGF family comprises the following seven members: VEGF-A, VEGF-B, VEGF-C, VEGF-D, VEGF-E, VEGF-F, and PlGF [30]. VEGF expression is reportedly inversely correlated with clinicopathological outcomes in ESCC [31, 32].

Our study demonstrated that GRP94 depletion inhibited cancer cell proliferation and metastatic potential by suppressing the AKT and MAPK pathways. In addition, we found that the reduction of IL-6, VEGF and COX-2 levels was due to suppression of NF-kB and AP-1 production after silencing GRP94 in ESCC cells. Therefore, we conclude that GRP94 may be a good therapeutic target for the treatment of ESCC.

## RESULTS

### Clinicopathological features of ESCC and GRP94 expression

**Figure 1:**
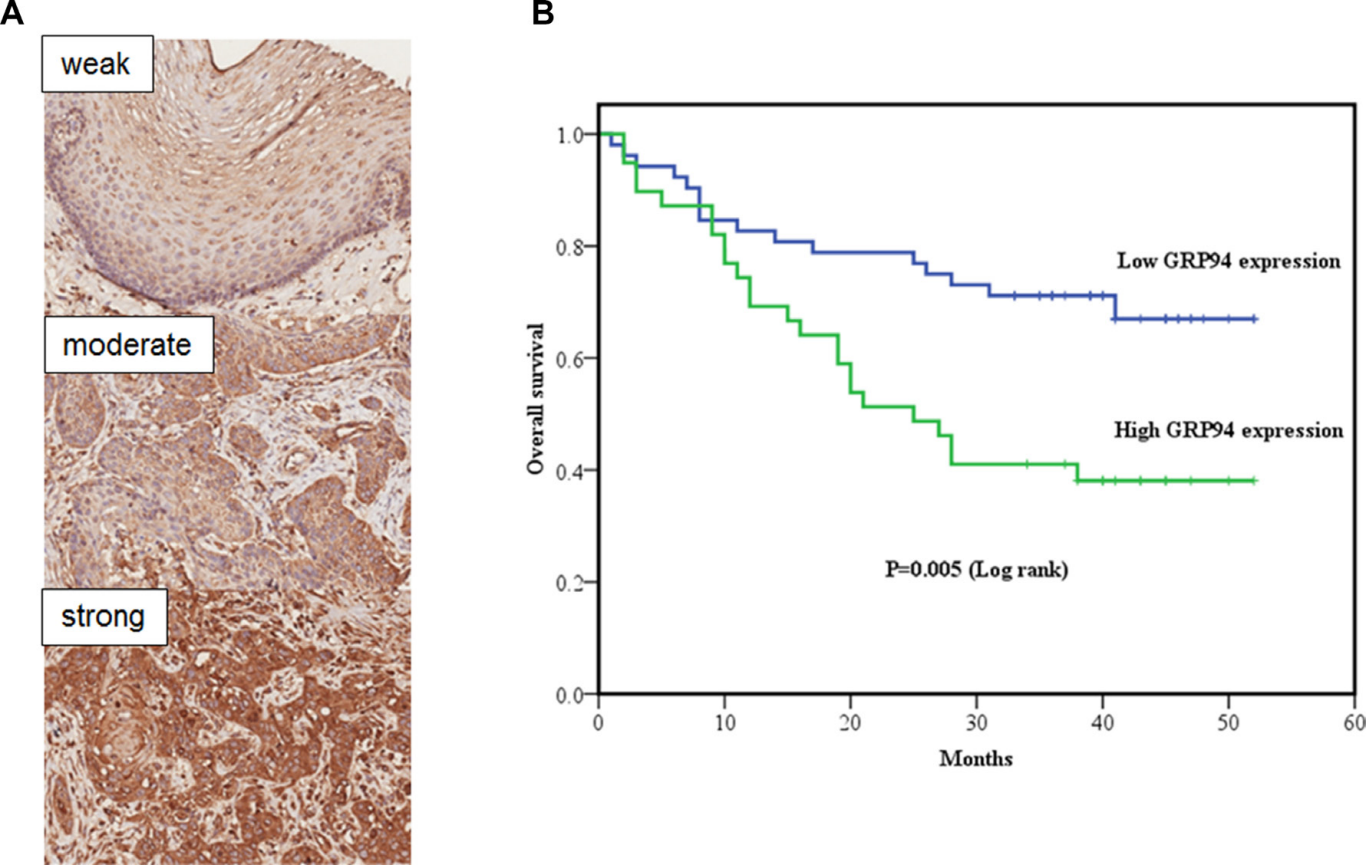
The clinical correlation of GRP94 in ESCC tissue arrays (**A**) GRP94 expression in normal esophageal squamous epithelium and squamous cell carcinoma (SCC). Upper: Focal and weak GRP94 expression in normal esophageal squamous epithelium. Middle: Focal and moderate GRP94 expression in esophageal SCC. Bottom: Diffuse and strong GRP94 expression in esophageal SCC (Original magnification, X200). We divided the GRP94 expression scores (0-300) into 2 groups: low expression (0-200) and high expression (201-300). (**B**) Kaplan-Meier curve of overall survival in esophageal squamous cell carcinomas (ESCC) with low GRP94 and high GRP94 expression. Statistical significance between the two groups (*P* = 0.005).

To better understand the prognostic role of GRP94 in ESCC, we performed immunohistochemical staining for GRP94 on ESCC tissue microarray sections. The clinicopathological features (HEso-Squ172Sur-02 tissue microarray) of the individuals who provided these samples are presented in Table 1. A total of 91 patients with clinicopathological and GRP94 expression data were evaluated. First, we compared GRP94 expression levels in ESCC tissue samples and matched normal esophageal squamous epithelial tissue samples (Figure 1A). ESCC tissue (GRP94 expression score: mean ± standard deviation, 201.6 ± 48.4) exhibited significantly higher GRP94 expression than normal esophageal squamous epithelial tissue (GRP94 expression score: 98.2 ± 50.0) (*P* < 0.001). The association between clinicopathological characteristics and GRP94 expression is presented in Table 1. Patients in the high GRP94 expression group tended to exhibit a higher frequency of lymph node metastasis than patients in the low GRP94 expression group (*P* = 0.032), and patients with high GRP94 expression levels tended to present at a later disease stage than patients with low GRP94 expression levels, although the difference between these two groups was not significant (*P* = 0.057).

**Table 1:** Association between clinicopathological characteristics and GRP94 expression

Characteristics	GRP94 expression		*P* value
	Low (*n* = 52)	High (*n* = 39)	
Gender			0.770
Male	40 (77%)	31 (79%)	
Female	12 (23%)	8 (21%)	
Mean Age (years) ± SD	63.3 ± 7.9	66.0 ± 9.2	0.129
Age			0.328
<65 years	32 (62%)	20 (51%)	
≥65 years	20 (38%)	19 (49%)	
Grading of SCC			0.327
Well differentiated	8 (15%)	6 (15%)	
Moderately differentiated	38 (73%)	24 (62%)	
Poorly differentiated	6 (12%)	9 (23%)	
Invasive depth of tumor^*1^			0.395
T1+T2	17 (34%)	10 (26%)	
T3+T4	33 (66%)	29 (74%)	
Lymph node metastasis^*2^			
Negative	35 (70%)	18 (47%)	0.032
Positive	15 (30%)	20 (53%)	
Stage (AJCC 7th Ed) ^*3^			0.057
I + II	37 (77%)	22 (58%)	
III + IV	11 (23%)	16 (42%)	

^*1^ Missing data in 2 cases; ^*2^ missing data in 3 cases; ^*3^ missing data in 5 cases

Abbreviation: AJCC, American Joint Committee on Cancer

### Analysis of the prognostic impact of GRP94 expression on overall survival

Kaplan-Meier curve analysis demonstrated that overall survival was significantly higher among patients with low GRP94 expression levels than among patients with high GRP94 expression levels (*P* = 0.005) (Figure 1B). Univariate and multivariate analyses were performed using Cox proportional hazards models to identify independent prognostic factors for overall survival (Table 2). Univariate analysis demonstrated that male gender, deeper invasion (T3+T4), lymph node metastasis, advanced pathologic stages (stages III and IV) and high GRP94 expression levels were associated with poorer prognosis. Multivariate analysis demonstrated that gender, age, and high GRP94 expression levels were independent prognostic factors for overall survival. Similar results were observed using the other tissue microarray (HEso-Squ172Sur-01) (data not shown).

**Table 2:** Univariate and multivariate analyses of clinicopathological factors and GRP94 expression affecting overall survival

Variables	Overall survival		*P* value
	Hazard ratio	95% CI	
*Univariate analysis*			
Gender			
Male	1	−	−
Female	0.334	0.119−0940.	0.038
Age			
< 65 years	1	−	−
≥ 65 years	1.549	0.832−2.883	0.167
Grading of SCC			
Well-moderately differentiated	1	−	−
Poorly differentiated	1.726	0.820−3.630	0.150
Invasive depth of tumor			
T1+T2	1	−	−
T3+T4	2.439	1.076−5.530	0.033
Lymph node metastasis			
Negative	1	−	−
Positive	1.895	1.002−3.582	0.049
Stage (AJCC 7th Ed)			
I + II	1	−	−
III + IV	2.215	1.159−4.250	0.017
GRP94 expression			
Low	1	−	−
High	2.480	1.276−4.546	0.007
*Multivariate analysis*			
Gender			
Male	1	−	−
Female	0.143	0.040−0.509	0.003
Age			
< 65 years	1	−	−
≥ 65 years	2.107	1.047−4.241	0.037
Grading of SCC			
Well-moderately differentiated	1	−	−
Poorly differentiated	1.941	0.836−4.505	0.123
Invasive depth of tumor			
T1+T2	1	−	−
T3+T4	2.140	0.746−6.144	0.157
Lymph node metastasis			
Negative	1	−	−
Positive	1.083	0.207−5.673	0.925
Stage (AJCC 7th Ed)			
I + II	1	−	−
III + IV	1.597	0.260−9.811	0.613
GRP94 expression			
Low	1	−	−
High	2.194	1.095−4.394	0.027

Abbreviations: CI, confidence interval; AJCC, American Joint Committee on Cancer.

### GRP94 expression analysis and manipulation in ESCC cells

**Figure 2:**
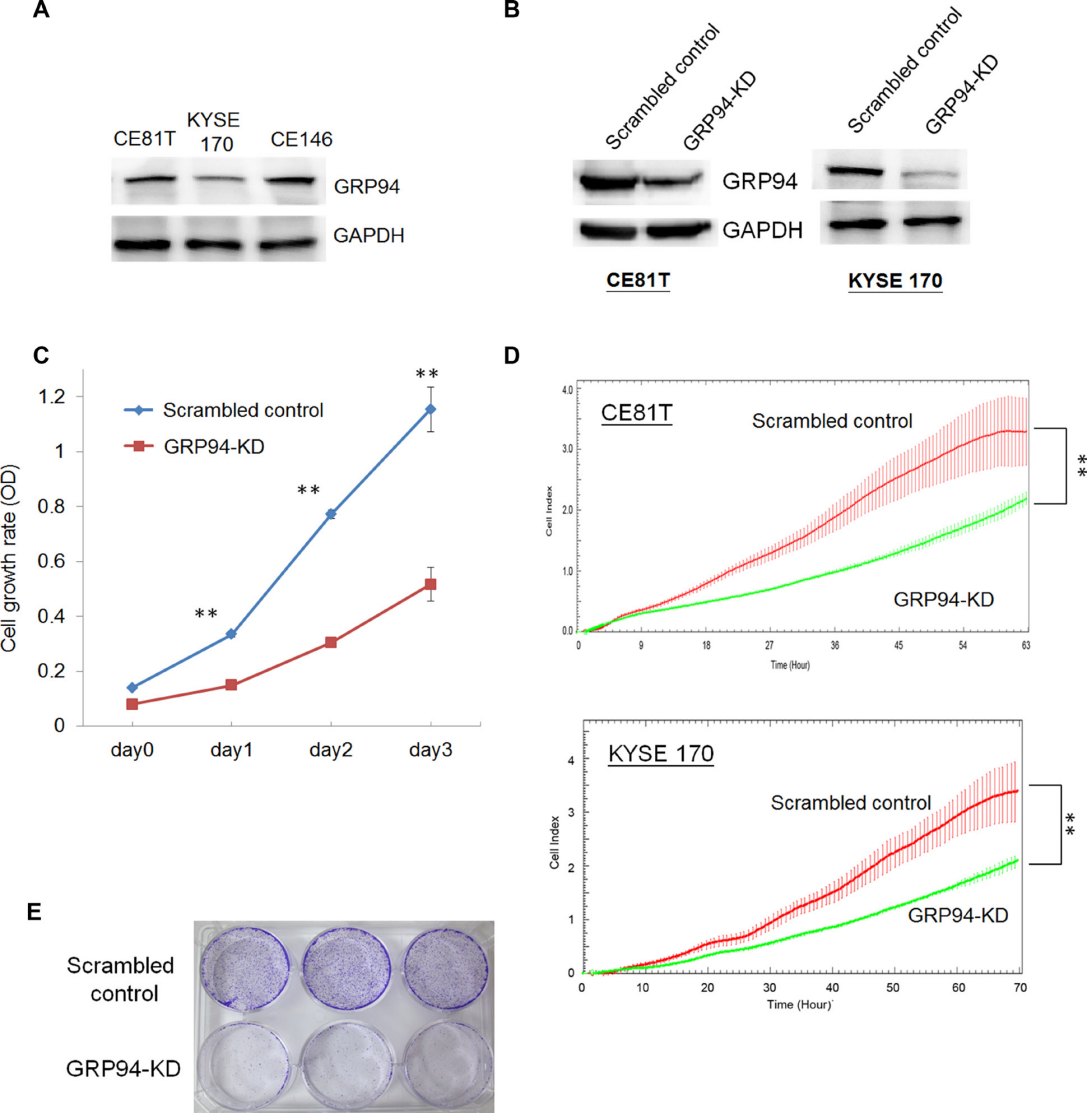
Silencing GRP94 reduced the proliferative activity in ESCC (**A**) The level of GRP94 in ESCC cells was determined by western blotting. GAPDH was the internal control. (**B**) The levels of GRP94 in scrambled control and GRP94-KD cells were determined. (**C**) The cell growth activity was determined by MTT assay. (**D**) The x’CELLigence system was applied to determine the proliferation of scrambled control and GRP94-KD CE81T and KYSE 170 cells. (**E**) Colony formation was performed using the scrambled control and GRP94-KD CE81T cells. All the experiments were repeated at least three times independently. ^**^ indicates *P* < 0.01.

To examine the role of GRP94 in ESCC, we assessed GRP94 expression in the indicated ESCC cell lines. As shown in Figure 2A, KYSE 170, CE81T, and CE146T cells exhibited highly GRP94 expression by western blotting,indicating that GRP94 plays an essential role in ESCC. To elucidate the role of GRP94 in ESCC further, we knocked down GRP94 expression using shRNA and determined the level of GRP94 in GRP94-shRNA transfected (GRP94-KD) CE81T and KYSE 170 cells (Figure 2B). The level of GRP94 was reduced dramatically in GRP94-KD CE81T and KYSE 170 cells compared with scrambled control shRNA-transfected (scrambled control) cells.

### Silencing GRP94 decreased cell proliferation

To analyze the biological effects of GRP94 down-regulation in ESCC cells, we assessed GRP94-KD and scrambled control CE81T cell growth via MTT assays and a biosensor system. GRP94-KD CE81T cells exhibited a lower growth rate than scrambled control CE81T cells (Figure 2C). Using the xCELLigence biosensor system, we also observed that GRP94-KD cell growth was reduced by more than 50% compared with scrambled control cell growth (Figure 2D). In the colony formation assay, GRP94-KD cells produced fewer colonies than scrambled control cells (Figure 2E). Overall, these results indicate that suppressing GRP94 expression in ESCC cells diminished their growth activity.

### Silencing GRP94 decreased ESCC metastasis and invasiveness

**Figure 3:**
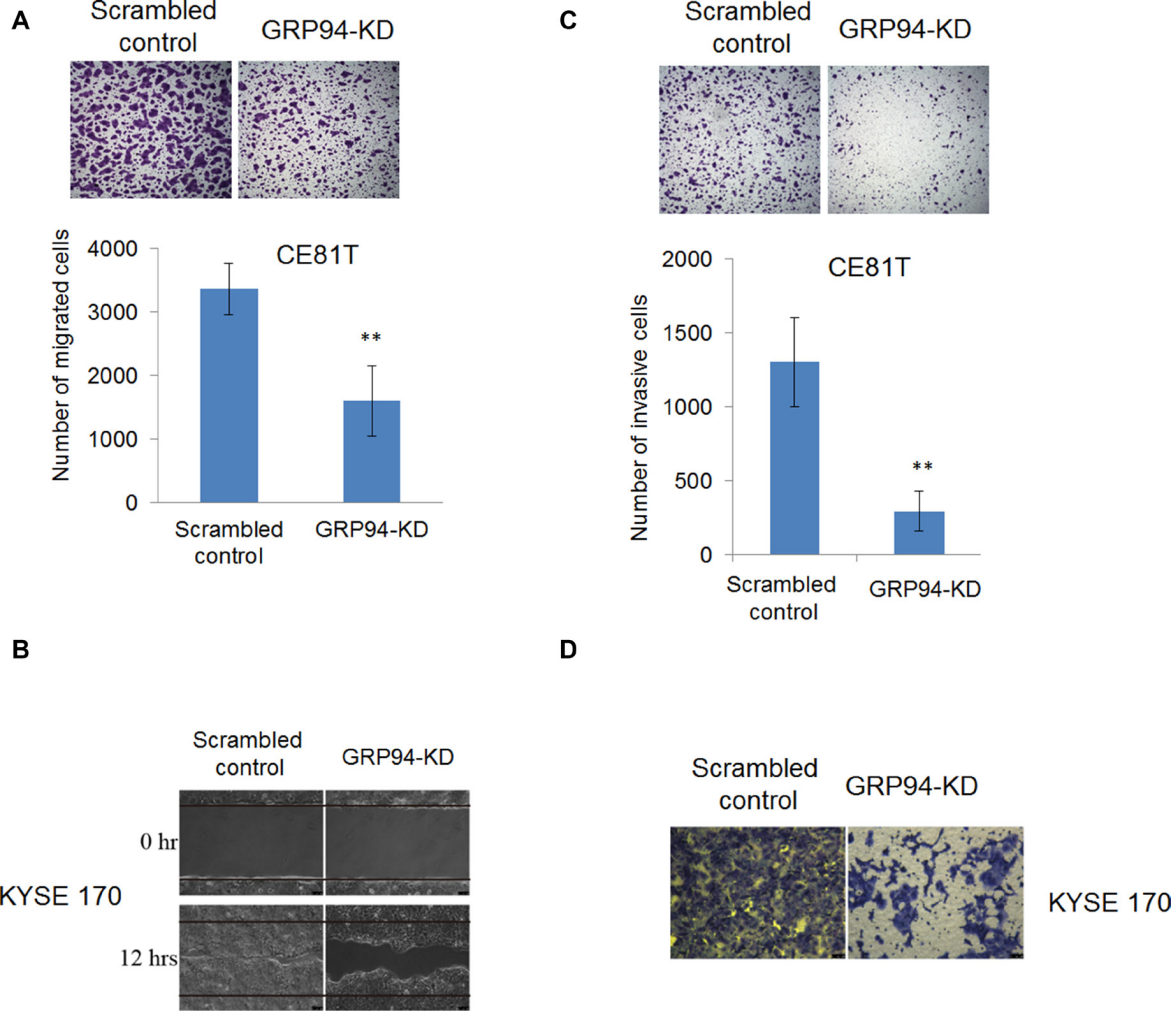
Silencing of GRP94 suppressed metastatic ability in ESCC cells (**A**) The migratory ability of scrambled control and GRP94-KD CE81T cells was determined by Transwell system. In wound-healing migratory assay, (**B**) GRP94-KD KYSE 170 cells showed a slower healing ability than scrambled control cells. (**C–D**) The invasiveness of scrambled control and GRP94-KD CE81T cells was determined by invasion assay. Silenced GRP94 showed the reduction of invasive ability in CE81T cells (C) and KYSE 170 cells (D). All the experiments were repeated at least three times independently. ^**^ indicates that *P* < 0.01.

Many ESCC patients present with stage III disease when first diagnosed with cancer, indicating that understanding the molecular mechanisms underlying ESCC metastasis is important and may facilitate the development of better therapeutic strategies for the treatment of ESCC. We examined the role of GRP94 in ESCC metastasis via transwell migration, wound-healing and invasion assays. As shown in Figure 3A, GRP94-KD CE81T cells exhibited less migration than scrambled control cells. In wound-healing migratory assay, silenced GRP94 in KYSE 170 cells caused a reduction of wound-healing ability compared with scrambled control cells (Figure 3B). Similarly, in invasion assays, more invasive cells were present in the scrambled control group than in the GRP94-KD group (Figure 3C and 3D). These results indicated that GRP94 mediated metastasis ability in ESCC cells.

### Silenced GRP94 suppressed proliferation in a zebrafish model

**Figure 4:**
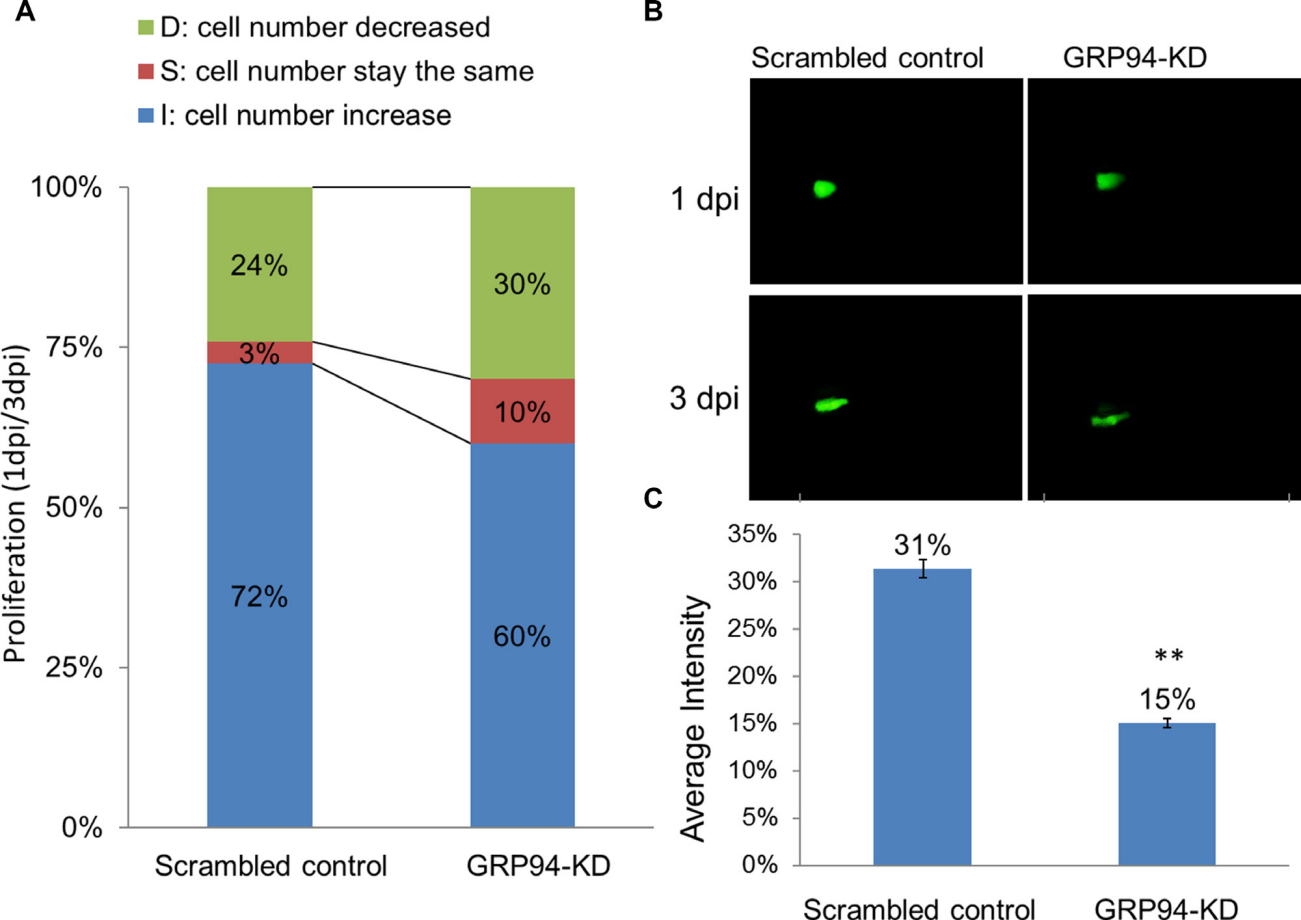
Silenced GRP94 suppressed proliferation in a zebrafish model (**A**) Scrambled control and GRP94-KD cells were injected into embryos. The embryos were checked for fluorescent cells at 2 h post-transplantation and were examined at one and three days post-injection (1 dpi and 3 dpi). Comparison of the 1 dpi vs. 3 dpi stages showed differences in proliferative activity between scrambled control and GRP94-KD cells. (**B**–**C**) The image of the embryo was obtained using an immunofluorescence microscope. The fluorescence intensity was quantified. The data are presented as the means of 6 experiments ± SD. ^**^P < 0.01.

To further confirm the role of GRP94 in ESCC progression, the xenotransplantation assay was performed in zebrafish system. In brief, scrambled control and GRP94-KD cells were implanted into the embryo yolk. As shown in Figure 4, we compared 1dpi vs. 3dpi stages to demonstrate the proliferative activity between scrambled control and GRP94-KD cells. The cell numbers increase embryo in scrambled control and GRPP94-KD was 72% vs 60%, indicating that silencing GRP94 caused a decrease in cell growth ability in CE81T cells.

### GRP94 was involved in the maintenance of mitochondrial bioenergetics

**Figure 5:**
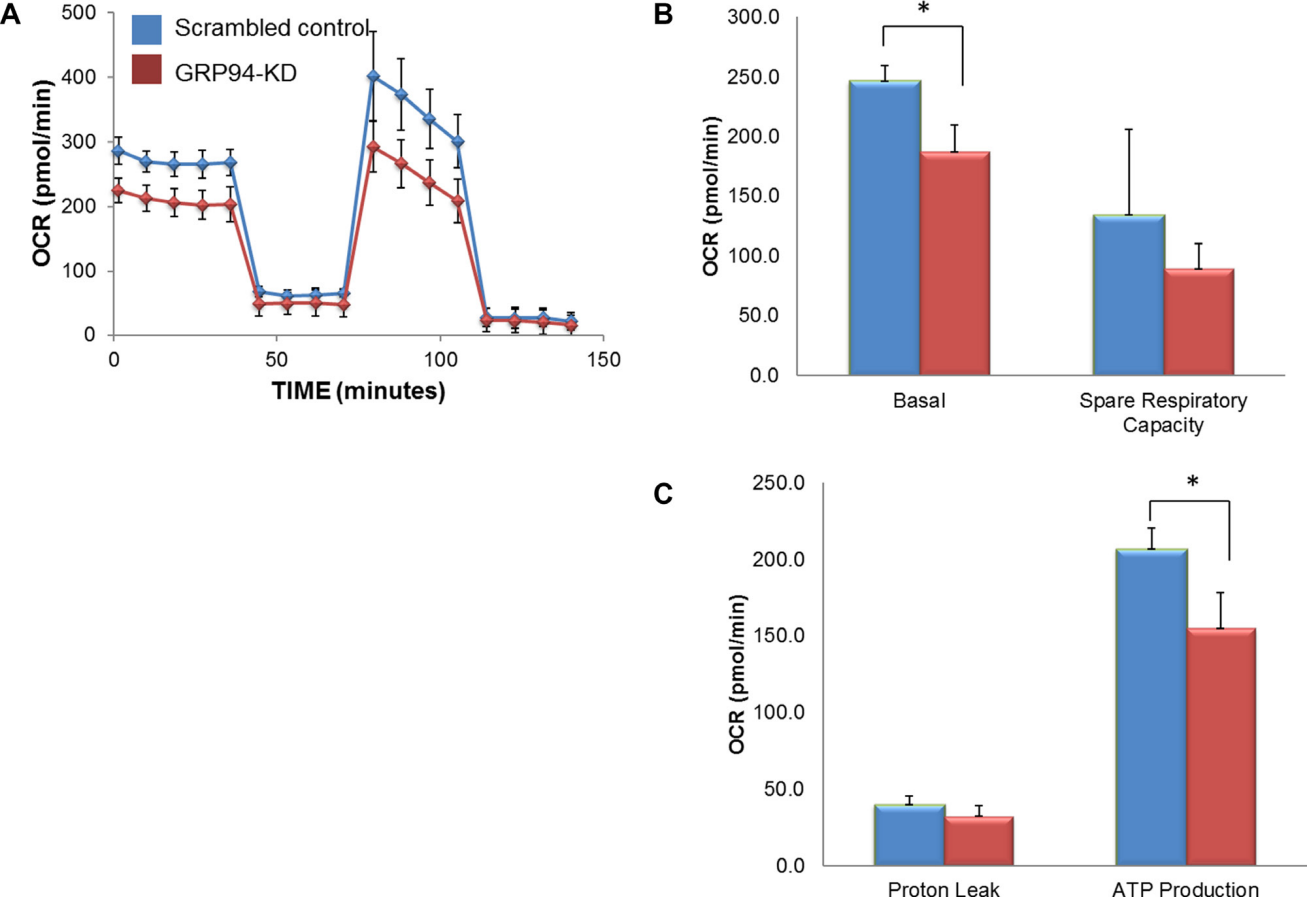
GRP94 silencing influenced mitochondrial respiration The cell metabolism of scrambled control and GRP94-KD CE81T cells was assessed using the XFe24 Analyzer. (**A**) Mitochondrial respiration was measured by the oxygen consumption rate (OCR). (**B–C**) After establishing the baseline, ATP production, maximal respiration, spare respiratory capacity and proton leak were measured following sequential addition of oligomycin (ATP synthase inhibitor), FCCP (a mitochondrial uncoupling agent) and rotenone (electron transporter channel inhibitor). Experiments were repeated three times with similar data trends, and the reported values are from a representative experiment. ^*^ indicates *P* < 0.05.

Mitochondria function and energy metabolism are key mediators of cancer progression and metastasis. To determine whether GRP94 might influence ESCC metabolism, intact cellular respiration was detected using the Seahorse XF24 Metabolic Flux Analyzer. The data shown in Figure 5 represent the time course of the OCR under basal conditions and following the sequential additions of oligomycin (ATP synthase inhibitor), FCCP (a mitochondrial uncoupling agent) and rotenone (electron transporter channel inhibitor). GRP94-KD cells exhibited significantly lower OCR, basal respiration, maximum respiration, and spare respiratory capacity than scrambled control cells. However, GRP94-KD cells exhibited no difference in ECAR or proton leakage compared to control cells (data not shown). Those results indicated that silencing GRP94 might influence mitochondrial bioenergetics in ESCC cells.

### GRP94 influenced mitochondrial structure

**Figure 6:**
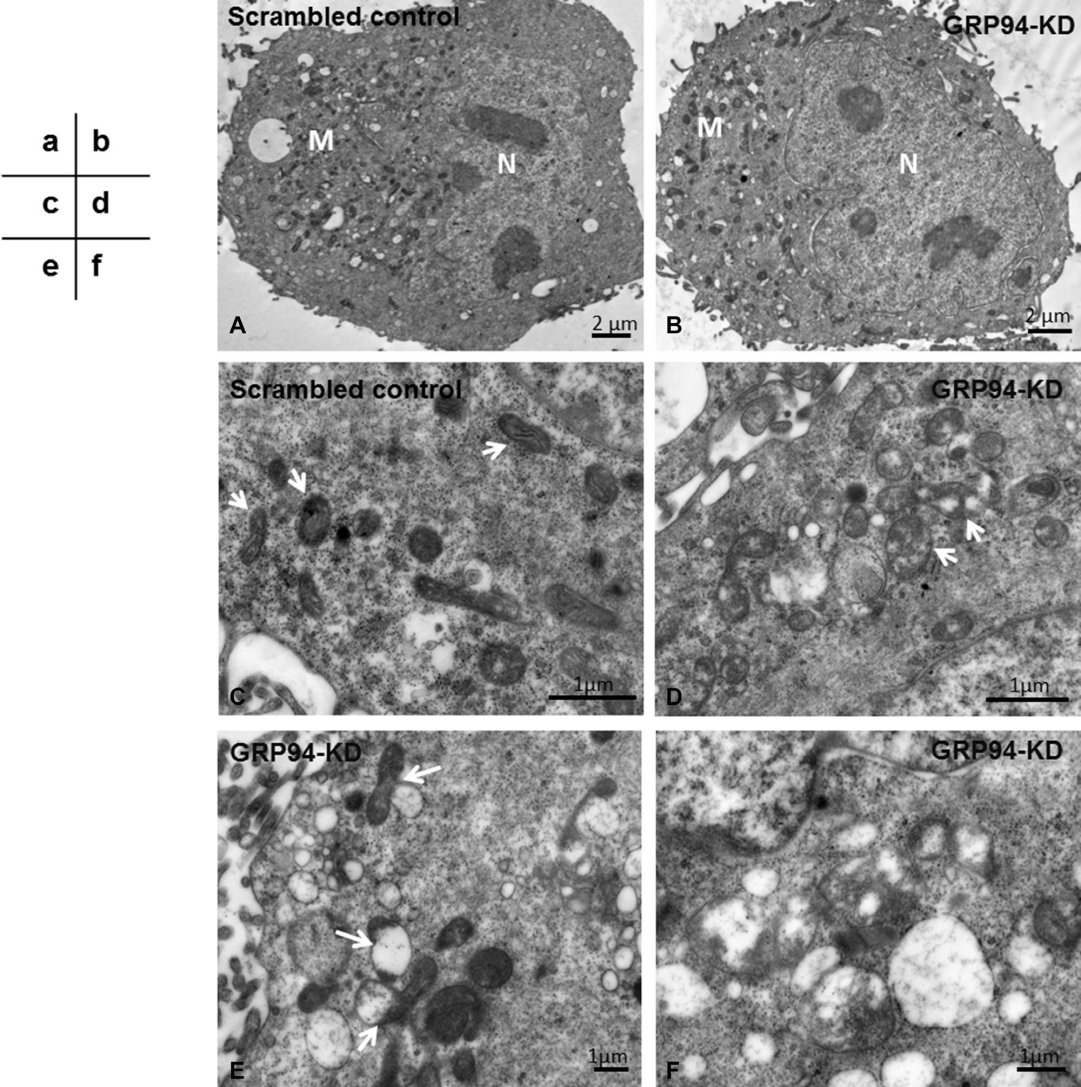
Silencing GRP94 promoted mitophagy (**A–B**) The mitochondria (M) were located in the cytoplasm outside of nuclei (N). Under low magnification, the numbers of mitochondria in the scrambled control and GRP94-KD CE81T cells were similar. (**C**) Under higher magnification, the mitochondria in scrambled control cells were clear and had well-defined cristae (arrows). (**D**) The mitochondria in GRP94-KD cells had lost a portion of the cristae, leaving translucent patches (arrows). The mitochondria in GRP94-KD cells had been degraded by autophagosomes. (**E**) The impaired mitochondria were attached to and digested by autophagosomes (arrows). (**F**) Higher magnification showing mitochondrial remnants engulfed by autophagosomes.

To further confirm the influence of GRP94 in mitochondria, transmission electron microscopy (TEM) was performed. Morphologically, both scrambled control cells and GRP94-KD cells exhibited intact plasma membranes encircling their cellular structures and regularly shaped nuclei within their cellular structures (Figure 6A–6B, N). The numbers of mitochondria in scrambled control and GRP94-KD cells were somewhat comparable (Figure 6A–6B, M). However, the interiors of the mitochondria were significantly different between scrambled control and GRP94-KD cells (Figure 6C–6D). The mitochondria in scrambled control cells exhibited clear and well-defined cristae, formed by inner membrane folding and the major sites of ATP generation. This observation indicated that mitochondrial function was not disrupted in these cells (Figure 6C, arrows). In contrast, the mitochondria in GRP94-KD cells exhibited missing cristae and several translucent patches where these cristae had been, indicating that mitochondrial function was impaired in these cells (Figure 6D, arrows). These damaged mitochondria were subsequently bound, engulfed and degraded by autophagosomes via mitophagy (Figure 6E, arrows). Higher magnification images revealed remnants of mitochondria in autophagosomes (Figure 6F). Those results suggest that silencing GRP94 might influence the stability of mitochondria in ESCC cells.

### Silencing GRP94 influenced AKT and ERK pathway activation

**Figure 7:**
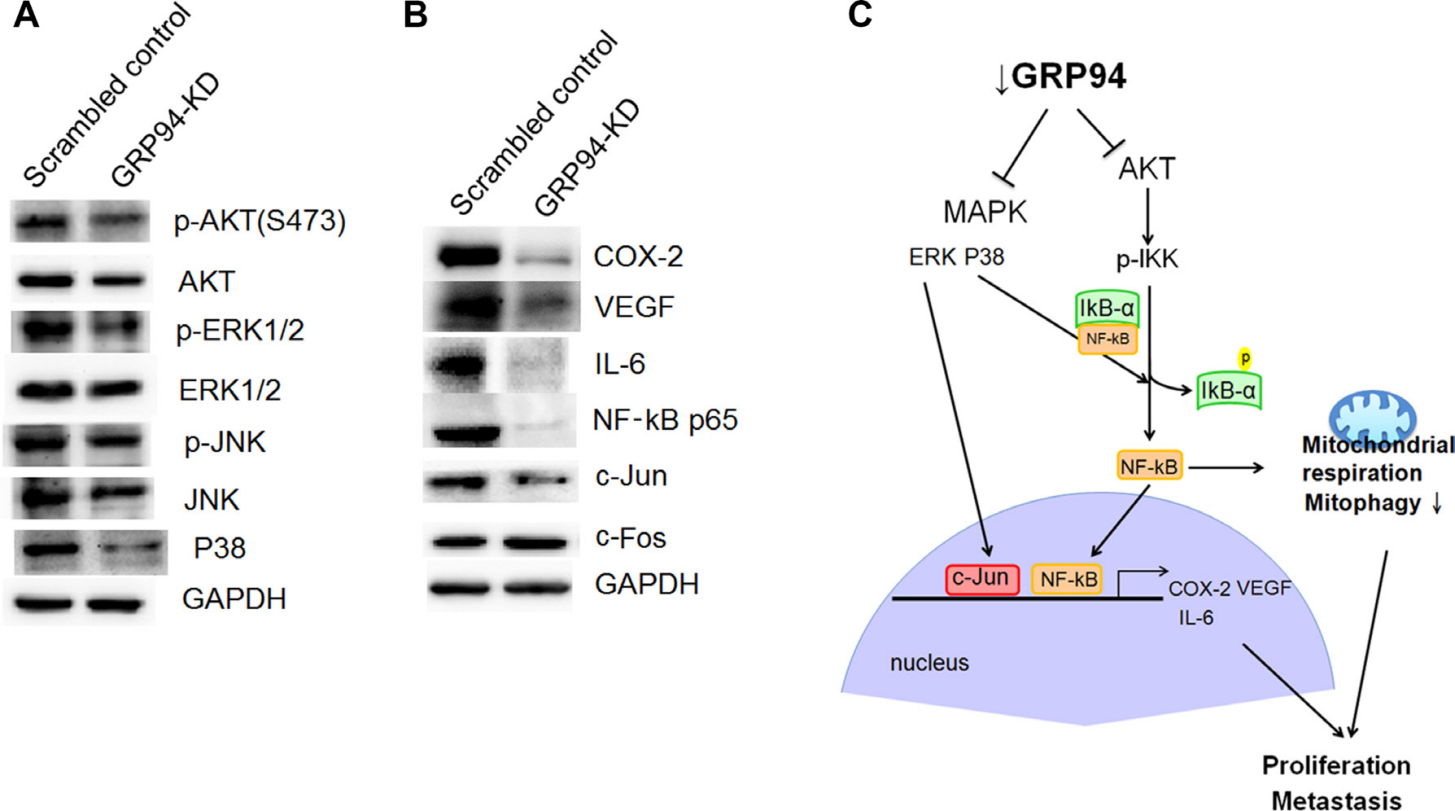
Silencing GRP94 suppressed the AKT and MAPK-mediated pathways (**A**) The levels of AKT, p-AKT, JNK, p-JNK, p38, ERK and p-ERK were determined. Activation of AKT and ERK was suppressed in GRP94-silenced cells. (**B**) The levels of COX-2, VEGF, IL-6, c-Jun, c-Fos, and NF-kB were determined by western blotting. (**C**) Schematic showing how silencing GRP94 may inhibit the AKT and MAPK pathways. The subsequent reduction of COX-2, IL-6, and VEGF may be due to the reduction of NF-kB activation and AP-1 production.

The AKT and ERK pathways are involved in cancer proliferation and metastasis. We assessed AKT, p-AKT, ERK, and p-ERK levels in scrambled control and GRP94-KD cells. Both AKT and p-AKT levels were reduced after GRP94 knockdown, as shown in Figure 7A. There was no difference in ERK levels between scrambled control and GRP94-KD cells; however, p-ERK levels were dramatically decreased in GRP94-KD cells compared with scrambled control cells. In contrast, JNK pathway activity was not affected by GRP94 knockdown. These findings suggested that GRP94 potentiated cell growth via the AKT and ERK pathways.

### Targeting GRP94 caused reductions in AP-1 levels and NF-kB mediated VEGF, IL6 and COX-2 levels

We next assessed AP-1 (c-Jun and c-Fos) and p38 expression and observed that c-Jun and p38 expression was dramatically suppressed in GRP94-KD cells compared with scrambled control cells (Figure 7B). c-Fos levels were unchanged by GRP94 knockdown. COX-2 is also a key molecular regulator of cancer progression and metastasis and has become an important target in cancer therapy. We assessed COX-2 levels in GRP94-KD and scrambled control cells and observed that down-regulation of GRP94 expression caused dramatic reductions in COX-2 levels, as shown in Figure 7B. This observation indicated that GRP94 might regulate COX-2 levels in ESCC cells to facilitate cancer progression and metastasis. We attempted to further determine the downstream target of COX-2. We found that silencing GRP94 caused reductions in VEGF and IL-6 expression in ESCC cells. To further elucidate the upstream regulator of COX-2, we assessed the level of NF-kB and found dramatically reduced the levels of NF-kB p65 (Figure 7B). Took together, these findings showed that silencing GRP94 might influence VEGF and IL-6 expression levels via the NF-kB/COX-2 axis.

### AKT and COX2 inhibitor treatments reduced the migration and invasion properties of ESCC cells

**Figure 8:**
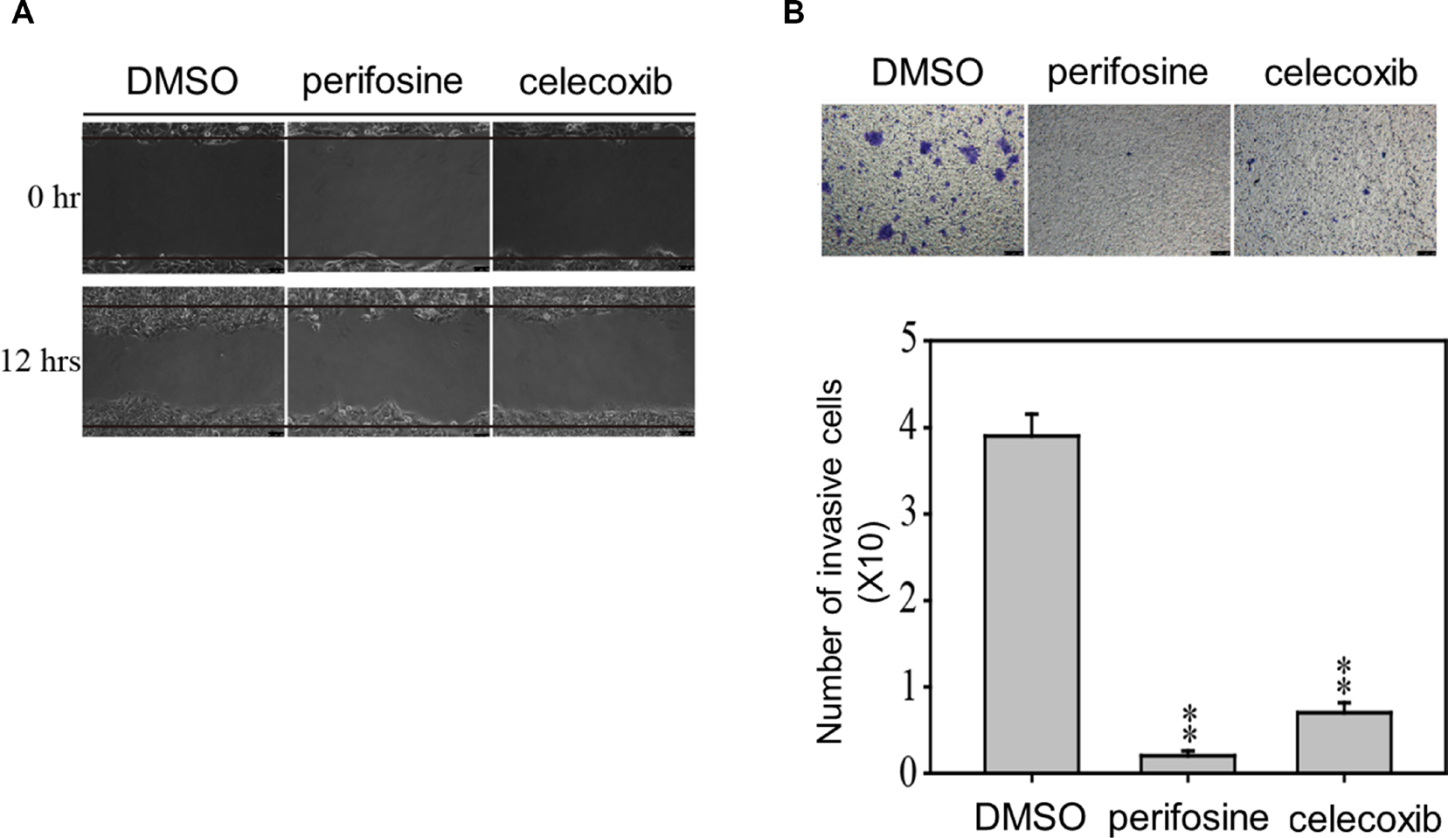
AKT and COX-2 inhibitors suppress the metastatic and invasion abilities of CE81T cells The migration (**A**) and invasion (**B**) abilities of CE81T cells in the presence of 10 μM AKT inhibitor (perifosine) or 10 μM COX-2 inhibitor (celecoxib) were determined by wound healing and transwell assays. In the invasion assay, the invaded cells were stained with 0.1% crystal violet and evaluated by microscopy. All experiments were repeated at least three times independently. ^**^ indicates *P* < 0.01.

To confirm the role of AKT and COX-2 in the migration and invasion ability of ESCC, we next accessed whether AKT and COX-2 would be important factors regulating migration and invasion properties of CE81T cells (Figure 8). We observed that treated with AKT inhibitor (perifosine, 10 μM) and COX-2 (celecoxib, 10 μM) to CE81T cells significantly inhibited migration and invasion at 12 and 48 h, indicating that GRP94 regulated AKT activation and COX-2 levels in ESCC cells to facilitate cancer progression and metastasis.

## DISCUSSION

In general, early diagnosis is the most important issue with respect to ESCC patient management. Most patients diagnosed with ESCC in Taiwan initially present with stage III disease; therefore, the establishment of a screening process to facilitate early ESCC diagnosis in affected patients is very important. Regarding ESCC treatment, radical esophagectomy with extensive lymph node dissection, chemoradiotherapy and photodynamic therapy improve the prognosis and quality of life of patients with non-metastatic ESCC [33–35]. However, treatment is much more complicated in ESCC patients with locally advanced, recurrent or metastatic disease. Esophageal cancer treatment is very challenging because the disease recurrence rate remains high, and recurrence is often accompanied by distant metastasis to the liver or lungs [36]. Specific prognostic or therapeutic biomarkers of esophageal cancer are therefore urgently needed.

GRPs are stress-inducible chaperones residing mainly in the ER and mitochondria. Previous studies have demonstrated that GRPs play specific roles in tumorigenesis [29, 37], progression and metastasis [38, 39]. Many reports have shown that GRPs are therapeutic targets for cancer treatment, and many different compounds targeting GRPs are being developed. Our previous study showed that GRP94-KD cervical cancer cells are more chemoresistant to docetaxel due to suppression of the mitochondrial caspase-mediated cell death pathway [16]. In both human ESCC and adenocarcinoma [27, 40], GRP94 protein expression levels are higher in cancer tissue than in adjacent normal mucosal tissue. The role of GRP94 in esophageal cancer proliferation and migration is not clear. We demonstrated a significant correlation between GRP94 expression and ESCC development and progression (Figure 1), as the results of our *in vitro* assays suggested that GRP94 depletion could inhibit cell growth and metastasis. We also observed that GRP94 mediated VEGF expression levels via the NF-kB/COX-2 axis (Figure 7). This is the first evidence of a regulatory relationship between GRP94 and NF-kB/COX-2 axis.

COX-2, an isoform of COX, catalyzes the conversion of arachidonic acid into inflammatory PGs, which play important roles in carcinogenesis. Extensive evidence gathered over the past several decades indicates that COX-2 expression is enhanced in many premalignant tissues and malignant tumors, including Barrett’s esophagus and esophageal cancer [41, 42]. Relatively high COX-2 expression levels have been observed in advanced-stage lung cancer with lymph node metastases [43], and further studies indicate that COX-2 overexpression is associated with angiogenesis [44] as well as tumor invasion and metastasis [45, 46]. COX-2 also modulates P-glycoprotein (MDR-1), which contributes to drug resistance [47]. COX-2-derived PGs play an important role in regulating esophageal tumor cell proliferation and apoptosis [48]. Thus, COX-2 inhibition may be useful in the treatment of esophageal cancer. Large-scale epidemiological studies have indicated that regular use of non-steroidal anti-inflammatory drugs, such as aspirin, which mainly acts by inhibiting COX-1 and COX-2, may reduce the risk of esophageal cancer [49], as selective COX-2 inhibition not only inhibits tumor invasiveness and metastasis in colon cancer cells [50] but also induces apoptosis in multidrug-resistant cell lines [51].

It is well-known that the energy requirements of rapidly growing tumor cells are met by predominantly glycolytic metabolism with high-energy requirements through the use of mitochondrial respiration [52]. In addition, increasing evidence suggests a strong association between the energy metabolism of tumor cells and tumor progression and development [53–55]. The glucose regulated protein, GRP94 is a stress-inducible molecular chaperone that is mainly localized in the cytosol and nucleus. GRP94 is also found in the endoplasmic reticulum (ER) and mitochondria, which are key organelles regulating protein quality control and metabolic balance, maintaining the integrity and homeostasis of the ER and mitochondria under physiological and pathological conditions [56–58]. Structural and functional analyses reveal zones of close contact between the ER and mitochondria [59]. Signaling from the ER to mitochondria can be critical in the induction of mitochondrial dependent cell death pathways [60]. Thus, the lack of GRP94 expression not only causes ER stress and downstream ER stress-responsive genes to bolster ER protein-folding capacity, but it also disturbs mitochondrial structural and functional characteristics to trigger mitochondrial dependent apoptosis. Accordingly, GRP94 overexpression is associated with lymph node metastasis and carcinoma recurrence in gastric carcinomas [20], whereas silencing of GRP94 dramatically inhibits cancer cell migration and proliferation abilities *in vitro* [61]. The present findings support previous reports showing that higher GRP94 expression levels are associated with lower overall survival and higher lympho-node metastasis, while silencing GRP94 impairs mitochondria by reducing basal respiration and ATP production in ESCC cells. GRP94 knockdown-induced ER stress dysregulation may be directly linked to mitochondria dysfunction in ESCC cells, resulting in suppression of cancer growth and metastatic potential.

Our findings indicate that GRP94 depletion caused a reduction in the expression of VEGF and its downstream target molecule COX-2, which might be the major factor causing suppression of tumor growth and metastasis. This is the first study to demonstrate that GRP94 influences IL-6 and VEGF expression in ESCC. To understand how GRP94 regulates VEGF expression, we conducted a miRNA analysis to determine whether GRP94 depletion suppresses VEGF expression by altering miRNA levels. Our results indicate that the level of miR107, but not miR125a, was up-regulated dramatically due to GRP94 depletion in ESCC (data not shown). However, additional experiments are necessary to determine how GRP94 influences miR107 levels. In conclusion, we observed that GRP94 depletion induces miR107 up-regulation and VEGF suppression in ESCC cells. Clinical biomarkers shown to be predictive of certain epithelial tumors, such as EGFR or KRAS mutations in colon cancer, are infrequent and/or not predictive of esophageal cancers [62]. The PI3K/AKT pathway plays a crucial role in endothelial cell proliferation and differentiation [63]. AKT is a client protein of HSP90, the functionality of which is required for AKT pathway-mediated transduction of growth factor signaling [64]. The current study further demonstrates that GRP94 depletion suppresses AKT and ERK pathway activation.

## MATERIALS AND METHODS

### Chemicals and reagents

Triton X-100, Tris–HCl, neomycin, Trypan blue, EDTA, ribonuclease-A, and dimethyl sulfoxide (DMSO) were obtained from Sigma Chemical Co. (St. Louis, MO, USA). AKT inhibitor (perifosine) and COX-2 (celecoxib) was purchased from Selleckchem (Huston, TX, USA). Antibodies against GRP94, c-Fos, c-Jun, NF-kB and GAPDH were purchased from Santa Cruz Biotechnology, Inc. (Santa Cruz, CA, USA). The anti-VEGF antibody was purchased from Millipore, and antibodies against JNK, p38, p-AKT, AKT, p-ERK, and ERK were purchased from Cell Signaling Technology. The anti-COX2 and p-JNK antibody were purchased from Abcam. The anti-IL6 antibody was purchased from Genescript.

### Cell culture

Human ESCC cells (CE81T and CE146T) were purchased from the Food Industry Research and Development Institute (Hsinchu City, Taiwan). KYSE 170 cell was a gift from Pro. Yi-Ching Wang (National Cheng Kung University) and Prof. Ruo-Kai Lin (Taipei Medical University). These cells were maintained in Dulbecco’s Modified Eagle Medium (DMEM) (Gibco BRL, Grand Island, NY, USA) supplemented with 10% fetal calf serum (Gibco BRL, Grand Island, NY, USA), 4 mM L-glutamine, 100 μM non-essential amino acids, and 2% penicillin–streptomycin (10,000 U/mL penicillin and 10 mg/mL streptomycin) in a humidified incubator containing 5% CO_2_ at 37°C, as previously described [65, 66].

### Tissue samples and immunohistochemistry

Two sets of ESCC tissue microarrays (catalog no. HEso-Squ172Sur-01 and HEso-Squ172Sur-02) were purchased from Shanghai Outdo Biotech Co. (Shanghai, China). The pathologic diagnoses of these cases were microscopically reconfirmed. The sections were deparaffinized, rehydrated, and blocked with 3% hydrogen peroxide. Heat-induced antigen retrieval was performed in citric acid buffer (pH 6.0) at 121°C for 10 min using a decloaking chamber (Biocare Medical, Concord, CA, USA), after which the sections were incubated with goat polyclonal GRP94 antibodies (catalog No.: sc1794, 1:600; Santa Cruz Biotechnology, Dallas, TX, USA) at 4°C overnight. Then, the sections were incubated with a biotin-conjugated rabbit anti-goat antibody (catalogue No.: AP106B, 1:500, Chemicon International, Billerica, MA, USA) at room temperature for 30 min before being incubated with a prediluted streptavidin-*horseradish peroxidase* complex (Dako, Glostrup, Denmark) at room temperature for 10 min. The antigens were revealed by addition of 3,3′-diaminobenzidine, followed by counterstaining with hematoxylin. Appropriate positive and negative controls were included in these assays. Both the intensity and the extent of GRP94 expression in carcinoma cells were evaluated and scored using a previously described method [67]. Cytoplasmic staining intensity was scored semiquantitatively as follows: 0 point, negative; 1 point, weakly positive; 2 points, moderately positive; and 3 points, strongly positive. The percentage of positive tumor cells (0–100%) was multiplied by the GRP94 staining intensity; therefore, the overall score ranged from 0 to 300. We subsequently divided the GRP94 expression scores (0–300) into the following 2 groups: a low expression (0–200) group and a high expression (201–300) group.

### GRP94-knockdown esophageal cell line generation

The method used to generate GRP94-knockdown (GRP94-KD) ESCC cells with small hairpin RNA (shRNA) was modified from that used in a previous report [24, 25]. GRP94-specific shRNA was purchased from the National RNAi Core Facility, Academia Sinica, Taiwan. The target mRNA sequence for the human GRP94 (NM_003299) gene was 5’-GCGAGACTCTTCAGCAACATA-3′ and the sequence of the non-target shRNA control vector (SHC002) was 5 ’-caacaagatgaagagcaccaa-3′ (Sigma Chemical Co., St. Louis, MO). The plasmids were transfected into ESCC cells using a Neon transfection system (Invitrogen Life Technologies, Grand Island, NY), as described previously [24, 25]. Stably transfected cells were selected using puromycin for two weeks, and GRP94 expression was verified by quantitative real-time PCR and western blotting.

### Protein extraction and western blot analysis

Cell lysates were prepared using cell lysis buffer containing protease inhibitors (Complete Protease Inhibitor Tablets, Boehringer Mannheim, Indianapolis, IN). The protein samples were separated by 10% SDS-PAGE under reducing conditions and electrotransferred onto PVDF membranes (Bio-Rad Laboratories). Immunoblotting was performed using specific antibodies, as described in the Figure legends, and horseradish peroxidase (HRP)-conjugated secondary antibodies (1:5000). The bands were visualized with enhanced chemiluminescence reagent (GE Healthcare, Piscataway, NJ) and detected using a VersaDoc 5000 imaging system (Bio-Rad Laboratories) [24, 25].

### Cell proliferation assays using an xCELLigence biosensor system

Experiments were conducted using an RTCA DP instrument (AECE Biosciences, Inc., San Diego, CA), as previously described [24, 25]. Growth curves were constructed using 16-well plates (E-plate 16, AECE Biosciences, Inc.). Cells were seeded in an E-plate 16 at a density of 10,000 cells/well in FCS-containing medium. The plate was subsequently assessed once every 30 s for 4 h and once every half h thereafter. The data were analyzed using RTCA software version 1.2 (supplied with the instrument).

### Cell viability assay

Cells were plated at a density of 2 × 10^4^ cells/well in 24-well plates and incubated overnight in a 37°C, 5% CO_2_ incubator. The medium was aspirated, the remaining cells were further incubated with 0.25 mg/mL MTT for 1 h and subsequently extracted with DMSO, and the color change in the extract was measured at 550 nm using a spectrophotometer.

### Colony formation

A total of 1000 cells were seeded per well in a 6-well plate and cultivated for 2 weeks. Subsequently, the cells were fixed and stained with crystal violet. Quantification of crystal violet staining were observed under a phase contract microscope.

### Transwell migration assay

*In vitro* cell migration was investigated using an 8-μm BD Falcon^TM^ culture insert (BD Biosciences), as previously described [66]. Specifically, 1×10^5^ cells were suspended in 500 μL of serum-free media and then seeded into the upper compartment of the chamber. The lower compartment was filled with 1 mL of 10% FCS-containing media. After 24 h of incubation, the migrated cells on the reverse side of the membrane were stained with 0.1% crystal violet, and cell images were captured using an Olympus IX71 inverted microscope (Olympus Corp., Tokyo, Japan), under which the cells were counted at 100-fold magnification.

### Invasion assay

*In vitro* cell invasion assay was performed using an 8-μm BD Falcon^TM^ cell culture insert (BD Biosciences) [68]. Aliquots of 1 × 10^5^ cells were seeded into the upper compartment of the chamber. The lower compartment was filled with DMEM medium supplemented with 10% FCS. After 24 h of incubation, the noninvasive cells were removed from the upper surface of the membrane, while the invasive cells were stained with 0.1% crystal violet. Cell images were captured using an Olympus IX71 inverted microscope (Olympus Corp., Tokyo, Japan), under which the cells were counted at 100-fold magnification.

### Seahorse XF24 metabolic flux analysis

Intact cellular respiration was detected using the Seahorse XF24 Metabolic Flux Analyzer (Seahorse Bioscience, Chicopee, MA, USA). The cells were cultured in XF24-well microplates coated with CELL-TAK (BD Biosciences). Baseline measurements were recorded before the addition of 3 μM oligomycin, 1 μM carbonyl cyanide-4-(trifluoromethoxy)phenylhydrazone (FCCP), and 1 μM rotenone. The oxygen consumption rate (OCR), extracellular acidification rate (ECAR), an indicator of lactic acid production or glycolysis), spare respiratory capacity, and proton leakage were automatically calculated and recorded using Seahorse XF24 software. The percent change from the basal rate was calculated as the change in the rate divided by the average baseline rate.

### Xenotransplantation

The assay was performed by Taiwan Zebrafish Core Facility - Human Disease Model Resource Center. In brief, at two-days post-fertilization (dpf), the zebrafish embryos were dechorionated and subsequently anesthetized with tricaine (0.04 mg/ml, Sigma, St. Louis, MO, USA). Scrambled control or GRP94-KD CE81T cells were harvested, and labeled with CM-Dil (red fluorescence) (Vybrant; Invitrogen, Carlsbad, CA, USA). Approximately 200 cells (4.6 nl) were implanted into the yolk of each 2-dpf embryo using a Nanoject II Auto-Nanoliter Injector (Drummond Scientific, Broomall, PA, USA). After injection, the zebrafish embryos were washed once with fish water and incubated for 1 h at 28°C. The embryos were checked for fluorescent cells at 2 h post-transplantation and were examined at one and three-days post-injection (1dpi and 3 dpi) by fluorescence microscopy.

### Transmission electron microscopy

The cells were fixed with 2% paraformaldehyde and 2% glutaraldehyde (Electron Microscopy Sciences, PA, USA) in 0.1 M cacodylate buffer overnight at 4°C, after which the cells were washed three times with 0.1 M cacodylate buffer for 10 min each. The cells were subsequently fixed with 1% OsO4 for 1h at RT and washed three times with 0.1 M cacodylate buffer for 10 min each. Dehydration was performed by incubating the samples in ascending EtOH concentrations of 50, 60, 70, 80, 90, 95, and 100% to completely remove residual water from the cells. The samples were then infiltrated with a mixture of absolute EtOH and Epon 812 resin (Electron Microscopy Sciences, PA, USA) at ratios of 3:1, 1:1, and 1:3 for 2 h at each step. Pure resin was then applied twice, first for 4 h and then overnight, with gentle agitation. The cells were then embedded in fresh pure resin and polymerized at 60°C overnight. Ultrathin sections were generated with a Leica EM UC6 ultramicrotome and contrasted with uranyl acetate and lead citrate. These sections were then viewed under a Hitachi H-7500 transmission electron microscope.

### Statistical analysis

Patient clinicopathological characteristics and GRP94 expression levels were compared using chi-square tests for categorical data and two-tailed Student’s t tests for continuous data. Overall survival curves were generated using the Kaplan-Meier method, and the difference in survival between the low GRP94 expression group and the high GRP94 expression group was evaluated via the log-rank test. A Cox proportional hazards model was utilized to identify clinicopathological factors significantly associated with patient prognosis. *P* < 0.05 was considered statistically significant. All statistical calculations were performed using SPSS Statistics 17.0 software (SPSS Inc. Chicago, IL, USA).
